# Exploring the impact of chronic obstructive pulmonary disease (COPD) on diabetes control in diabetes patients: a prospective observational study in general practice

**DOI:** 10.1038/npjpcrm.2015.32

**Published:** 2015-04-23

**Authors:** Hilde D Luijks, Wim JC de Grauw, Jacobus HJ Bor, Chris van Weel, Antoine LM Lagro-Janssen, Marion CJ Biermans, Tjard R Schermer

**Affiliations:** 1 Department of Primary and Community Care, Radboud university medical center, Nijmegen, The Netherlands; 2 Australian Primary Health Care Research Institute, Australian National University, Canberra, ACT, Australia

## Abstract

**Background:**

Little is known about the association between COPD and diabetes control parameters.

**Aims:**

To explore the association between comorbid COPD and longitudinal glycaemic control (HbA_1C_) and systolic blood pressure (SBP) in a primary care cohort of diabetes patients.

**Methods:**

This is a prospective cohort study of type 2 diabetes patients in the Netherlands. In a mixed model analysis, we tested differences in the 5-year longitudinal development of HbA_1C_ and SBP according to COPD comorbidity (present/absent). We corrected for relevant covariates. In subgroup effect analyses, we tested whether potential differences between diabetes patients with/without COPD were modified by age, sex, socio-economic status (SES) and body mass index (BMI).

**Results:**

We analysed 610 diabetes patients. A total of 63 patients (10.3%) had comorbid COPD. The presence of COPD was not significantly associated with the longitudinal development of HbA_1C_ (*P*=0.54) or SBP (*P*=0.33), but subgroup effect analyses showed significant effect modification by SES (*P*<0.01) and BMI (*P*=0.03) on SBP. Diabetes patients without COPD had a flat SBP trend over time, with higher values in patients with a high BMI. For diabetes patients with COPD, SBP gradually increased over time in the middle- and high-SES groups, and it decreased over time in those in the low-SES group.

**Conclusions:**

The longitudinal development of HbA_1C_ was not significantly associated with comorbid COPD in diabetes patients. The course of SBP in diabetes patients with COPD is significantly associated with SES (not BMI) in contrast to those without COPD. Comorbid COPD was associated with longitudinal diabetes control parameters, but it has complex interactions with other patient characteristics. Further research is needed.

## Introduction

Chronic obstructive pulmonary disease (COPD) is typically diagnosed in middle-aged subjects who also have an increased risk for other chronic conditions.^1^ The presence of other diseases in addition to an ‘index disease’ is defined as comorbidity.^2^ Among patients with mild-to-moderate COPD, the main causes of death are comorbid diseases such as lung cancer and cardiovascular diseases.^3^ COPD has a large impact on morbidity and mortality.^4^

Another example of a chronic disease with marked effects on global health and health care is type 2 diabetes.^4,5^ Of all patients with COPD, 9–13% of the patients have comorbid diabetes,^6–9^ and 4–13% of patients with diabetes have comorbid COPD.^9–11^ Although these numbers originate from different studies and consequently are not directly comparable, they clearly illustrate that the combination of COPD and diabetes is a rather common one.

In recent years, knowledge and awareness of the importance of patient-specific factors in the treatment of COPD^12^ and diabetes^13^ has grown, resulting in an increased tendency to individualise disease management. An important characteristic of a patient with a specific chronic disease, such as COPD, is the comorbidity that may also be present. However, current guidelines for COPD and diabetes have limited applicability for patients with comorbid conditions.^14^ Very little is known on how the presence of a specific disease, such as COPD, influences the long-term outcomes of another disease, such as diabetes. This type of information is important if health care professionals aim to personalise disease management plans for COPD and diabetes patients. In addition, other characteristics such as age, sex, body mass index (BMI) and socio-economic status (SES) may have well-known effects on COPD^15–17^ and on diabetes,^18,19^ but how they interact if both diseases are present in one and the same patient is unknown. Detailed data on comorbidity, patient characteristics and disease control parameters from a representative patient population may inform us about the interaction between the two diseases and the impact on patients’ prognosis.

The aim of this explorative, hypothesis-generating paper was to investigate the association between COPD as a comorbid condition and longitudinal diabetes control parameters in patients with type 2 diabetes in primary care. We also explored the role of sex, age, BMI and SES in the relationship between COPD and diabetes control.

## Materials and methods

### Design and study subjects

We used available data from a dynamic prospective cohort of diabetes patients registered in the Continuous Morbidity Registration (CMR), a general practice registration network in the Nijmegen region, the Netherlands. The four practices constituting the CMR have been recording all morbidities that are presented to the general practitioners (GPs) on a daily basis since 1967.^20^ The database reflects the health care system in the Netherlands,^21^ where patients are registered with a general practice and have access to specialist care through that practice. In this system, where GPs receive capitation payment, the nature of medical conditions or treatment does not influence the GPs’ performances. Details on the composition of our diabetes cohort are described elsewhere.^10^ In short, we included all adult patients with a new diagnosis of type 2 diabetes within the observation period of the study (1 January 1985 to 31 December 2006). Time from the start to the end of observation varied between patients, beginning with either the start of the study period or the date of a patient’s enrolment in a CMR practice. The observation period ended either at the end of our study period or with a patient’s death or deregistration from the practice.

All four CMR practices also participate in the so-called ‘Nijmegen Monitoring Project’ (NMP),^22^ which was initiated in 1985 to systematically record diagnostic and monitoring measurements of patients with diabetes and/or hypertension. The NMP database includes demographic data, physical diagnostics (e.g., blood pressure, weight, height) and laboratory data (e.g., HbA_1C_, glucose levels). Monitoring data are collected by the GPs and practice nurses during routine 3-monthly diabetes check-up visits for all diabetes patients under GP care. The practices involved have been shown to provide good-quality diabetes care.^22^ We linked data from these two databases to study the effects of chronic comorbidity (data originating from the CMR) on the course of diabetes control over time (data from the NMP). The current paper presents results from the effect of COPD, as selected comorbid disease, on longitudinal diabetes control parameters, and effect modification by a number of patient characteristics in subgroup effect analyses. This analysis is part of a larger project studying the effects of different types of comorbid diseases on diabetes control parameters.

The CMR and NMP registries comply with the Code of Conduct for Health Research, which has been approved by the Data Protection Authorities for conformity with the applicable Dutch privacy legislation. For this study, approval of an ethics committee was not required.

### Selection of COPD and other comorbidities

The presence of COPD was identified as a doctor diagnosis recorded in the CMR database. The CMR has previously been used to study cohorts of patients with COPD,^23,24^ and the diagnoses correlate well with spirometry results.^25^ Details on the recording of comorbidity have been reported in a previous paper.^10^ We selected comorbid COPD as a single disease of particular interest for the analysis of possible associations between comorbid conditions and the course of diabetes control parameters, and we were, in addition, interested especially in comorbid malignancies and cardiovascular, mental and musculoskeletal diseases.

### Study outcomes

HbA_1C_ (in %, the current unit during our study period) and systolic blood pressure (SBP, in mmHg) were the primary study outcomes. Measurement of HbA_1C_ is performed at the annual check-up visits. Blood pressure measurement is generally performed at every check-up visit. To include patients with sufficient follow-up starting from the diagnosis, we only included patients with their first measurement performed within the first 4 months after the diabetes diagnosis and labelled these as ‘baseline measurements’. All subsequent measurements were regarded as repeated measurements for individual patients and contributed to the longitudinal analysis. We studied the development of these outcomes during the 5-year follow-up.

### Statistical analysis

SPSS (version 20.0) and SAS (version 9.02) software supported the analysis. Characteristics of the study population are provided using descriptive statistics. We compared linear trends for both HbA_1C_ and SBP in the 5 years after the diabetes diagnosis between patients with and patients without comorbid COPD. We applied a random intercept mixed model analysis using measurements nested within patients.^26^ In this model, the presence of existing COPD, i.e., recorded before the diabetes diagnosis, was the variable of interest. We added an interaction term ‘time’ by ‘COPD’ (absent/present) to the model to explore differences in HbA_1C_ and SBP trends according to the absence or presence of COPD. In this comparison between patients with and without COPD, we entered sex, age at diabetes diagnosis, SES, BMI (handled as ‘last observation carried forward’^26^) and the presence of other comorbidities (as specified above) as potential confounders. Values for age and BMI were handled as continuous variables in the mixed model, but we categorised them as ‘low’, ‘intermediate’ and ‘high’ values to facilitate (graphical) presentation of the results. The categorisation was based on the limits of the first, second (i.e., the median) and third quartiles of the distribution of age and BMI values of the patients who contributed to the analysis.

Furthermore, we performed subgroup effect analyses to test whether potential differences in the HbA_1C_ and SBP trends between diabetes patients with or without comorbid COPD were modified by sex, age, SES or BMI. The confounders in the initial analysis were now tested for potential effect modification separately by adding an interaction term ‘time’ by ‘COPD’ (absent/present) by ‘potential effect modifier’ to the model. Nonsignificant interaction terms were removed in a stepwise backward elimination procedure.^26^ In these subgroup effect analyses, we added the presence of other comorbidities as potential confounders (not as potential effect modifiers). In cases in which no significant results arose from the subgroup effect analysis, the first model (without subgroup effect analysis) defined the results.

Not only comorbid COPD already present at the study start may be associated with the longitudinal diabetes outcomes, the same may be the case for incident COPD after the patient’s diabetes diagnosis. Therefore, we performed sensitivity analyses excluding the patients who did not have COPD at their diabetes diagnosis date but who were diagnosed with COPD during the 5-year follow-up.

A *P*-value <0.05 was considered statistically significant.

## Results

### Study subjects and baseline characteristics

**Figure 1:**
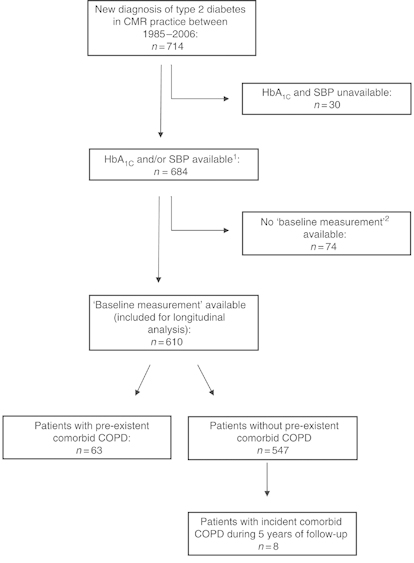
^1^Patients with the GP responsible for diabetes treatment. ^2^A patient’s first outcome measurements collected from a diabetes check-up visit within the first 4 months since the diabetes diagnosis was labelled as ‘baseline measurement’. CMR, Continuous Morbidity Registration; COPD, chronic obstructive pulmonary disease; GP, general practitioner; SBP, systolic blood pressure.

Figure 1 shows a flowchart of our study population. We included 610 patients with a mean age of 63 years (s.d. 12.5, Table 1) for longitudinal analysis. In all, 63 patients (10.3%) had comorbid COPD at the date of their diabetes diagnosis, and another 8 patients were diagnosed with COPD during the 5-year follow-up period. Patients with pre-existing COPD were older and had more additional comorbid conditions, apart from COPD, compared with patients without COPD (i.e., musculoskeletal disease, 51 vs. 30%). Note that in the longitudinal analyses we corrected for the presence of selected comorbidity.

### Influence of comorbid COPD on the course of HbA_1_c and SBP

**Figure 2:**
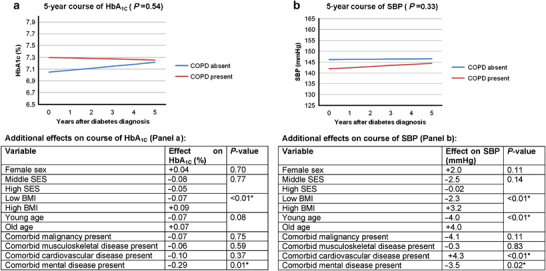
Mixed model results (no subgroup effect analysis): longitudinal HbA_1C_ (**a**) and SBP (**b**) outcomes of diabetes patients with and without comorbid COPD. Comorbid diseases: absence and presence are assessed on the date of diabetes diagnosis. Number (*n*) of cases with completed longitudinal analysis (no missing data on any of the variables included in the mixed model throughout): 582. Cases with missing values for BMI: *n*=23, cases with missing values for SES: *n*=5. *P-values <0.05. Age and BMI categories: based on the distribution of age and BMI values of patients contributing to the analyses, limits for ‘low’, ‘intermediate’ and ‘high’ values were 54, 64 and 72 years for age, and 26.0, 28.5 and 31.8 kg/m^2^ for BMI, respectively. Graphs for ‘reference categories’: in the graphic presentation, graph lines represent HbA_1_C or SBP courses for specific patient variables—for example, a male patient from the low-SES group with a specific age and BMI. We define the (theoretical) combination of the patient characteristics ‘male sex, low SES, median age, median BMI and absence of other comorbidity’ as ‘reference category’. The ‘Additional effects table’ below each graph contains information needed to construct lines of predicted outcomes, based on the mixed model results, for other subjects than the ‘reference category’. It shows the additional effects (to be added to the graphs displayed above) for other covariates included in the model. These values are not time dependent and not dependent on the absence or presence of COPD. Example: HbA_1_C courses over time for patients with and without comorbid COPD are shown in **a**. The ‘Additional effects table’ shows an additional effect of +0.04 (% HbA_1C_) for female sex. This means 0.04 should be added to the blue line for female patients without COPD and 0.04 should be added to the red line for female patients with COPD. The *P*-value of 0.70 shows that this additional effect of sex on HbA_1C_ in this analysis is not statistically significant. BMI, body mass index; COPD, chronic obstructive pulmonary disease; SBP, systolic blood pressure; SES, socio-economic status.

After correction for covariates, comorbid COPD was not significantly associated with the course of HbA_1C_ (*P*=0.54) or SBP (*P*=0.33) values over time in the initial analyses. Figure 2 shows the time trends for patients with and without comorbid COPD and the additional effects of covariates. The figure footnotes provide information for the definition of the ‘reference category’.

**Figure 3:**
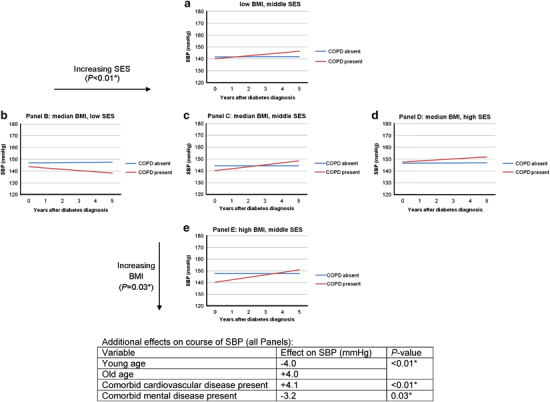
Mixed model results for subgroup effect analysis: 5-year course of SBP for diabetes patients with and without comorbid COPD, modified by SES (*P*<0.01) and BMI (*P*=0.03). The explanations are the same as Figure 2. Graphs are shown for the ‘reference category’ (i.e., male sex, median age, absence of other comorbidity), but SES and BMI vary as specified in the figure. BMI, body mass index; COPD, chronic obstructive pulmonary disease; SBP, systolic blood pressure; SES, socio-economic status.

In the subgroup effect analyses, however, we found a statistically significant association between comorbid COPD and the course of SBP, with effect modification of SES (*P*<0.01) and BMI (*P*=0.03). To express these complex findings in a comprehensible way, Figure 3 shows a graphical representation of the direction of effects, with separate graphs for combinations of SES and BMI. The figure shows that in the absence of COPD (blue lines), longitudinal SBP values are relatively stable over time, with higher values when BMI is higher (compare panels a, c and e). Diabetes patients with comorbid COPD (red lines) showed a more variable course of SBP over time, with SES more than BMI defining the direction of effects and absolute SBP values. Note that, in the subgroup effect analysis, nonsignificant terms were removed from the model; i.e., all variables presented contributed significantly to the model predicting the outcome. Age (*P*<0.01), presence of comorbid mental (*P*=0.03) and comorbid cardiovascular disease (*P*<0.01) had additional effects on the subgroup effect analysis results (not dependent on the presence or absence of comorbid COPD, additional effects). Absolute values depended on the mix of patient characteristics. No significant effect modification was found from any of the defined subgroups on the longitudinal development of HbA_1C_ in the presence of comorbid COPD.

### Sensitivity analysis

After exclusion of cases with incident COPD during the 5-year follow-up period, we did not observe a significantly different association between COPD and HbA_1C_ (*P*=0.54) or SBP (*P*=0.34), nor did we observe significant differences in the results from the subgroup effect analyses.

## Discussion

### Main findings

In the current study, we explored the association between comorbid COPD and the longitudinal development of HbA_1C_ and SBP in a representative cohort of newly diagnosed type 2 diabetes patients in primary care during 5 years of follow-up. The initial analyses showed no significant associations between COPD and these outcomes, but subgroup effect analysis indicated that, in the presence of COPD, the development of SBP was different for patients from different SES and BMI subgroups. This suggests that comorbid COPD, in relation with these particular patient characteristics, may influence long-term diabetes control parameters.

### Strengths and limitations of this study

In this dynamic cohort study, we used data from robust datasets that originate from decades of experience in morbidity recording in a practice-based research network from our department^20^ and good quality of diabetes care.^22^ We studied relevant diabetes control parameters as study outcomes (not ‘treatment intensification’)^27,28^ over a follow-up period that was long enough to assess potential associations with comorbid COPD. Comparison of outcomes over time between patients was meaningful, as we included only newly diagnosed diabetes patients. Another strength is that we studied an unselected population with ‘real patients’ receiving regular primary health care, i.e., a representative sample of the type 2 diabetes patient population.

It is important to realise that, within our study period, the criteria for the diagnosis of COPD and diabetes have changed. The current criteria for diagnosing COPD were introduced in Dutch general practice in 2001.^29^ Towards the end of our observation period, there was a higher rate of diabetes diagnoses.^30^ This implies that COPD and diabetes data from early in the observation period may not be fully comparable to similar data at the end of the period. This type of limitation is inherent to working with longitudinal data. In general, the CMR has shown to record diagnoses with high validity.^31^

One limitation of this study is that we were unable to account for smoking in the analyses, because this has not been consistently recorded in the CMR and NMP databases, nor did we have data available on the severity of COPD (i.e., degree of airflow obstruction, exacerbation rate, severity of dyspnoea). From a previous study, we know that the majority of COPD patients in the NMP registry have mild or moderate COPD,^25^ but from our current work we cannot tell whether and how the severity of underlying COPD may be associated with the course of diabetes outcomes.

Clearly, the development of HbA_1C_ and SBP over time as observed will have been influenced by the diabetes treatment as provided by GPs. This treatment may include stimulation of physical exercise (which is beneficial not only for the diabetes but also for the COPD) and prescription of glucose-lowering medication.^32^ Medication prescribed for COPD (e.g., oral or inhaled corticosteroids) may increase the glucose level and SBP in patients with diabetes.^33,34^ In this dynamic cohort study, it was not possible to compare therapeutic regimes between diabetes patients with and without COPD. Differences in medication or lifestyle regimes may have contributed to the observed differences.

Twenty-eight cases with missing data for SES or BMI throughout the follow-up period (variables included in the linear model) dropped out. Their numbers were relatively low, which makes it unlikely that they introduced bias.

The percentage of diabetes patients with comorbid COPD in our cohort corresponds with prevalence numbers described in the literature.^9–11^ Although the absolute number of patients with COPD (*n*=63) was relatively low, one of the subgroup effect analyses did show significant results. In a larger sample of diabetes patients with comorbid COPD, it would have been possible that some nonsignificant trends observed would have reached statistical significance.

The current paper is one result of a larger project with an explorative design aimed at investigating associations between several types of comorbidities on diabetes control parameters. These results generate new hypotheses and may guide further research elaborating on the early findings. It helps increase the evidence base for the complex care to patients with multimorbidity. We believe that the most important strength of the current work is precisely this novelty. To the best of our knowledge, this is the first study exploring longitudinal associations between COPD and another common chronic disease, in this case diabetes. Because the combined occurrence of diabetes and COPD is common, assessing possible interactions in terms of long-term outcomes is important. Our observation that, in some subgroups, comorbid COPD was associated with altered diabetes outcomes warrants further research in this area. Our study may serve as an example of how to investigate the complex relationships between two or more chronic conditions (i.e., multimorbidity) on patients’ prognoses for the diseases involved.

### Interpretation of findings in relation to previously published work

The unfavourable effect of increasing BMI on systolic blood pressure is not surprising.^35^ Our observations indicate that for diabetes patients with comorbid COPD, patient characteristics that predict long-term outcomes may be different from those without COPD. Our study design had an explorative nature in which we tested several associations; hence, care needs to be taken in the interpretation of our findings. We did not find significant associations between comorbid COPD and all study outcomes tested. It is possible that the significant associations between longitudinal SBP and comorbid COPD among diabetes patients may not be replicated in a future study. It cannot be concluded from observational research only whether and how our findings should be translated into therapeutic consequences. One could reason that, in patients with diabetes and comorbid COPD, factors related to a patient’s SES are more important in achieving long-term SBP control than just reducing BMI. Our finding that among COPD patients the lower SES group had the best long-term SBP control is surprising, but this finding would first need to be confirmed in a larger study before we should speculate about possible explanations. In our cohort, diabetes patients with comorbid COPD had different (comorbidity) characteristics than those without COPD—an important notion for the treatment of patients with either or both of these diseases. The observed differences may result from disease-specific or from generic factors such as obesity, lifestyle and smoking. The need for more research aiming at profoundly investigating the associations between COPD, SES and diabetes control parameters is obvious. Previous studies described negative associations between low SES and COPD^36^ and diabetes^19^ prognosis. Studies reporting on the relationship between SES and prevalence of multimorbidity in general described negative associations.^37–39^ We have not been able to trace any previously published papers paying attention to the role of SES in relation to the specific combination of COPD and diabetes.

After the first recognition of the importance of lipid regulation in diabetes,^40^ the revised version of the Dutch College of General Practitioners diabetes guideline in 1999 resulted in increased attention to the role of lipids halfway through our study period. For this reason, and as studying one glycaemic and one non-glycaemic control parameter already resulted in a large data set with many associations tested, we decided not to include lipids as diabetes control parameters.

Some covariates showed significant additional effects, both on the nonsignificant results from the initial models and on the significant subgroup effect analysis results. Note that these are independent from time and from the presence/absence of COPD. Augmenting effects from increasing BMI and age (on both study outcomes) and from comorbid cardiovascular disease (on SBP) can be expected among diabetes patients. We assume that the consistent diminishing effect of comorbid mental diseases is related to a higher consultation frequency among these patients,^41^ offering more opportunities to diagnose and manage diabetes (or hypertension) in an early stage, resulting in slightly better outcomes.

Pathophysiologic mechanisms that have been suggested to have a role in the relationship between respiratory impairment in COPD patients and diabetes include an increased BMI, altered respiratory compliance, weakness of the respiratory muscles or neuropathies.^7^

GPs’ beliefs about the feasibility and benefits of medication regimes may be influenced by the presence of comorbidity,^42–44^ which might result in deliberate flexible medication prescriptions in patients with comorbidities. Given these considerations, we believe that the absence of an association between comorbid COPD and long-term HbA_1C_ outcomes among diabetes patients implies that GPs responsible for treatment provide good-quality diabetes care despite the presence of comorbidities such as COPD.

### Implications for future research, policy and practice

The current study provides novel observational data in a research area that is still underdeveloped, i.e., multimorbidity. It focuses on the impact of COPD as a comorbid disease in patients with diabetes. Comorbidity should be regarded as a patient characteristic that may influence relevant outcomes of another disease. Instead of focussing mainly on disease-specific outcomes, future research should pay more attention to the effects of comorbidity and other patient characteristics such as socio-demographic background.

Moreover, future work may study the effects of (other) incident comorbidity on diabetes outcomes in more detail. Further investigations of potential associations between diabetes, and other prevalent chronic diseases, with relevant COPD outcomes are desired too, as well as other combinations of diseases.

The majority of practitioners caring for patients with either COPD or diabetes will see several patients with these diseases combined, and our findings may help raise awareness on the importance of formulating personalised management plans that aim for sensible outcomes taking into account both diseases. The current explorations do not yet allow for concrete recommendations for daily practice changes—our findings need to be replicated in larger diabetes cohorts.

Knowledge of the impact of comorbidity on disease outcomes is also important to support pay-for-performance initiatives that facilitate patient-centred care. Therefore, ongoing research in this area should be prioritised by funding bodies and policymakers.

### Conclusions

Comorbid COPD was associated with longitudinal control parameters of newly diagnosed type 2 diabetes patients in general practice. This association was observed on SBP (but not on HbA_1C_) and was modified by SES and BMI. Although these results need to be verified first, this exploratory study provides new information on the interaction between multiple chronic diseases, and may guide further development of personalised care that accounts for patients’ comorbidity.

## Figures and Tables

**Table 1:** Baseline characteristics of patient population, according to the presence/absence of COPD

	*Total included* n*=610*	*COPD present*^a^ n*=63*	*COPD absent*^a^ n*=547*	P*-value*^a^
*Patient characteristics*				
Sex: male, *n* (%)	294 (48.2)	34 (54.0)	260 (47.5)	0.33
Age at DM diagnosis, years; mean (s.d.)	63.0 (12.5)	69.0 (11.0)	62.3 (12.5)	<0.001
Follow-up time, years; mean (s.d., range)	6.2 (4.6; 0.1–21.9)	4.1 (3.6; 0.3–15.6)	6.5 (4.7; 0.1–21.9)	<0.001
Measurements per patient, total number, mean (median; s.d.; range)	21.8 (17.5; 18.2; 1–106)	15.7 (10; 14.9; 1–86)	22.5 (19; 18.4; 1–106)	0.001
BMI at baseline^b^, mean (s.d.), kg/m^2^	29.8 (5.1)	29.5 (5.2)	29.8 (5.1)	0.57
SES^b^, *n* (%)				
Low	315 (52.1)	37 (58.7)	278 (51.3)	0.26
Middle	242 (40.0)	24 (38.1)	218 (40.2)	
High	48 (7.9)	2 (3.2)	46 (8.5)	
Year of diabetes diagnosis, *n* (%)				
1985–1989	83 (13.6)	3 (4.8)	80 (14.6)	0.07
1990–1999	235 (38.5)	24 (38.1)	211 (38.6)	
2000–2006	292 (47.9)	36 (57.1)	256 (46.8)	
				
*Comorbidity data*				
Comorbid diseases^c^, mean number (s.d.; range)	2.7 (2.3; 0–11)	4.0 (2.5; 0–11)	2.6 (2.2; 0–11)	<0.001
Comorbid diseases^c^, (categorised), *n* (%)				
0	100 (16.4)	4 (6.3)	96 (17.6)	0.002
1 or 2	227 (37.2)	17 (27.0)	210 (38.4)	
3 and more	283 (46.4)	42 (66.7)	241 (44.1)	
Cardiovascular comorbidity^c^, present, *n* (%)	390 (63.9)	44 (69.8)	346 (63.3)	0.30
Musculoskeletal comorbidity^c^, present, *n* (%)	197 (32.3)	32 (50.8)	165 (30.2)	0.001
Mental comorbidity^c^, present, *n* (%)	140 (23.0)	18 (28.6)	122 (22.3)	0.26
Comorbid malignancy^c^, present, *n* (%)	42 (6.9)	4 (6.3)	38 (6.9)	0.86
Incident comorbid COPD after DM diagnosis^d^, *n* (%)	12 (2.0)	NA	12 (2.2)	NA
Incident COPD in the first year after DM diagnosis, *n* (%)	1 (0.2)	NA	1 (0.2)	NA
Incident COPD in the first 5 years after DM diagnosis, *n* (%)	8 (1.3)	NA	8 (1.5)	NA

Characteristics of patients at baseline, i.e., at the date of the diabetes diagnosis.

Abbreviations: BMI, body mass index; DM, type 2 diabetes mellitus; NA, not applicable; SES, socio-economic status.

a COPD presence/absence: assessed on the date of diabetes diagnosis. *P*-values displayed are calculated for the difference between the group with versus without comorbid COPD. We performed Chi-square tests for continuous variables and independent *t*-tests for continuous variables. *P*-values <0.05 were considered statistically significant.

b Number of measurements available for BMI at baseline: 576 (missing at baseline: *n*=34). Number of measurements available for SES at baseline: 605 (missing at baseline: *n*=5).

c Presence of any type of comorbid disease was assessed at the date of diabetes diagnosis. For the diabetes patients with comorbid COPD present at the diabetes diagnosis date, we excluded COPD in the count of the total number of comorbid diseases to make a meaningful comparison with the total number of comorbid diseases in patients without COPD.

d Mean time (after the diabetes diagnosis date) until the date of comorbid COPD diagnosis, for incident cases, is 4.6 years.

## References

[bib1] GOLD, Global Initiative for Chronic Obstructive Lung Disease . [10 November 2014] www.goldcopd.com .

[bib2] Van den Akker M Buntinx F Knottnerus J Comorbidity or multimorbidity: what's in a name? A review of literature Eur J Gen Pract 1996 2 65 70

[bib3] Sin DD Anthonisen NR Soriano JB Agusti AG Mortality in COPD: role of comorbidities Eur Respir J 2006 28 1245 1257 17138679 10.1183/09031936.00133805

[bib4] Lopez AD Mathers CD Ezzati M Jamison DT Murray CJ Global and regional burden of disease and risk factors, 2001: systematic analysis of population health data Lancet 2006 367 1747 1757 16731270 10.1016/S0140-6736(06)68770-9

[bib5] Van Dieren S Beulens JW Van der Schouw YT Grobbee DE Neal B The global burden of diabetes and its complications: an emerging pandemic Eur J Cardiovasc Prev Rehabil 2010 17 S3 S8 20489418 10.1097/01.hjr.0000368191.86614.5a

[bib6] Feary JR Rodrigues LC Smith CJ Hubbard RB Gibson JE Prevalence of major comorbidities in subjects with COPD and incidence of myocardial infarction and stroke: a comprehensive analysis using data from primary care Thorax 2010 65 956 962 20871122 10.1136/thx.2009.128082

[bib7] Mannino DM Thorn D Swensen A Holguin F Prevalence and outcomes of diabetes, hypertension and cardiovascular disease in COPD Eur Respir J 2008 32 962 969 18579551 10.1183/09031936.00012408

[bib8] Baty F Putora PM Isenring B Blum T Brutsche M Comorbidities and burden of COPD: a population based case-control study PLoS ONE 2013 8 e63285 23691009 10.1371/journal.pone.0063285 PMC3656944

[bib9] Barnett K Mercer SW Norbury M Watt G Wyke S Guthrie B Epidemiology of multimorbidity and implications for health care, research, and medical education: a cross-sectional study Lancet 2012 380 37 43 22579043 10.1016/S0140-6736(12)60240-2

[bib10] Luijks H Schermer T Bor H Van Weel C Lagro-Janssen T Biermans M Prevalence and incidence density rates of chronic comorbidity in type 2 diabetes patients: an exploratory cohort study BMC Med 2012 10 128 23106808 10.1186/1741-7015-10-128 PMC3523042

[bib11] Niefeld MR Braunstein JB Wu AW Saudek CD Weller WE Anderson GF Preventable hospitalization among elderly Medicare beneficiaries with type 2 diabetes Diabetes Care 2003 26 1344 1349 12716786 10.2337/diacare.26.5.1344

[bib12] Miravitlles M Soler-Cataluna JJ Calle M Soriano JB Treatment of COPD by clinical phenotypes: putting old evidence into clinical practice Eur Respir J 2013 41 1252 1256 23060631 10.1183/09031936.00118912

[bib13] Raz I Riddle MC Rosenstock J Buse JB Inzucchi SE Home PD Personalized management of hyperglycemia in type 2 diabetes: reflections from a Diabetes Care Editors' Expert Forum Diabetes Care 2013 36 1779 1788 23704680 10.2337/dc13-0512 PMC3661796

[bib14] Lugtenberg M Burgers JS Clancy C Westert GP Schneider EC Current guidelines have limited applicability to patients with comorbid conditions: a systematic analysis of evidence-based guidelines PLoS ONE 2011 6 e25987 22028802 10.1371/journal.pone.0025987 PMC3197602

[bib15] Roche N Deslee G Caillaud D Brinchault G Court-Fortune I Nesme-Meyer P Impact of gender on COPD expression in a real-life cohort Respir Res 2014 15 20 24533770 10.1186/1465-9921-15-20 PMC3931914

[bib16] Cao C Wang R Wang J Bunjhoo H Xu Y Xiong W Body mass index and mortality in chronic obstructive pulmonary disease: a meta-analysis PLoS ONE 2012 7 e43892 22937118 10.1371/journal.pone.0043892 PMC3427325

[bib17] Eisner MD Blanc PD Omachi TA Yelin EH Sidney S Katz PP Socioeconomic status, race and COPD health outcomes J Epidemiol Community Health 2011 65 26 34 19854747 10.1136/jech.2009.089722 PMC3017471

[bib18] Peters SA Huxley RR Woodward M Diabetes as risk factor for incident coronary heart disease in women compared with men: a systematic review and meta-analysis of 64 cohorts including 858,507 individuals and 28,203 coronary events Diabetologia 2014 57 1542 1551 24859435 10.1007/s00125-014-3260-6

[bib19] Grintsova O Maier W Mielck A Inequalities in health care among patients with type 2 diabetes by individual socio-economic status (SES) and regional deprivation: a systematic literature review Int J Equity Health 2014 13 43 24889694 10.1186/1475-9276-13-43 PMC4055912

[bib20] Van Weel C The Continuous Morbidity Registration Nijmegen: background and history of a Dutch general practice database Eur J Gen Pract 2008 14 (Suppl 1) 5 12 18949638 10.1080/13814780802436028

[bib21] Van Weel C Schers H Timmermans A Health care in the Netherlands J Am Board Fam Med 2012 25 (Suppl 1) S12 S17 22403245 10.3122/jabfm.2012.02.110212

[bib22] De Grauw WJ Van Gerwen WH Van de Lisdonk EH Van den Hoogen HJ Van den Bosch WJ Van Weel C Outcomes of audit-enhanced monitoring of patients with type 2 diabetes J Fam Pract 2002 51 459 464 12019056

[bib23] Van den Bemt L Schermer T Bor H Smink R Van Weel-Baumgarten E Lucassen P The risk for depression comorbidity in patients with COPD Chest 2009 135 108 114 18689578 10.1378/chest.08-0965

[bib24] Bischoff EW Schermer TR Bor H Brown P Van Weel C Van den Bosch WJ Trends in COPD prevalence and exacerbation rates in Dutch primary care Br J Gen Pract 2009 59 927 933 19891824 10.3399/bjgp09X473079 PMC2784530

[bib25] Hoogendoorn M Feenstra TL Schermer TR Hesselink AE Rutten-van Molken MP Severity distribution of chronic obstructive pulmonary disease (COPD) in Dutch general practice Respir Med 2006 100 83 86 15894477 10.1016/j.rmed.2005.04.004

[bib26] Twisk JWR Applied Longitudinal Data Analysis for Epidemiology1st edn. Cambridge University PressCambridge, UK, 2003

[bib27] Voorham J Haaijer-Ruskamp FM Wolffenbuttel BH De Zeeuw D Stolk RP Denig P Differential effects of comorbidity on antihypertensive and glucose-regulating treatment in diabetes mellitus—a cohort study PLoS ONE 2012 7 e38707 22679516 10.1371/journal.pone.0038707 PMC3367971

[bib28] Woodard LD Urech T Landrum CR Wang D Petersen LA Impact of comorbidity type on measures of quality for diabetes care Med Care 2011 49 605 610 21422952 10.1097/MLR.0b013e31820f0ed0 PMC3366691

[bib29] Geijer RMM TH Smeele IJM Sachs APE Bottema BJAM Van Hensbergen W Van Schayk CP NHG-Standaard COPD en Astma bij Volwassenen: Diagnostiek Huisarts Wet 2001 44 107 117

[bib30] Klein Woolthuis EP De Grauw WJ Van Gerwen WH Van den Hoogen HJ Van de Lisdonk EH Metsemakers JF Yield of opportunistic targeted screening for type 2 diabetes in primary care: the diabscreen study Ann Fam Med 2009 7 422 430 19752470 10.1370/afm.997 PMC2746521

[bib31] Van Weel C Validating long term morbidity recording J Epidemiol Community Health 1995 49 29 32 7561667 10.1136/jech.49.suppl_1.29 PMC1060866

[bib32] American Diabetes Association Standards of medical care in diabetes—2013 Diabetes Care 2013 36 (Suppl 1) S11 S66 23264422 10.2337/dc13-S011 PMC3537269

[bib33] Suissa S Kezouh A Ernst P Inhaled corticosteroids and the risks of diabetes onset and progression Am J Med 2010 123 1001 1006 20870201 10.1016/j.amjmed.2010.06.019

[bib34] Walters JA Walters EH Wood-Baker R Oral corticosteroids for stable chronic obstructive pulmonary disease Cochrane Database Syst Rev 2005 10.1002/14651858.CD005374 PMC13634668 16034972

[bib35] Souto-Gallardo Mde L Bacardi Gascon M Jimenez Cruz A Effect of weight loss on metabolic control in people with type 2 diabetes mellitus: systematic review Nutr Hosp 2011 26 1242 1249 22411367 10.1590/S0212-16112011000600008

[bib36] Lange P Marott JL Vestbo J Ingebrigtsen TS Nordestgaard BG Socioeconomic status and prognosis of COPD in Denmark COPD 2014 11 431 437 24568315 10.3109/15412555.2013.869580

[bib37] McLean G Gunn J Wyke S Guthrie B Watt GC Blane DN The influence of socioeconomic deprivation on multimorbidity at different ages: a cross-sectional study Br J Gen Pract 2014 64 e440 e447 24982497 10.3399/bjgp14X680545 PMC4073730

[bib38] Schafer I Hansen H Schon G Hofels S Altiner A Dahlhaus A The influence of age, gender and socio-economic status on multimorbidity patterns in primary care. First results from the multicare cohort study BMC Health Serv Res 2012 12 89 22471952 10.1186/1472-6963-12-89 PMC3348059

[bib39] Tucker-Seeley RD Li Y Sorensen G Subramanian SV Lifecourse socioeconomic circumstances and multimorbidity among older adults BMC Public Health 2011 11 313 21569558 10.1186/1471-2458-11-313 PMC3118239

[bib40] Pyorala K Pedersen TR Kjekshus J Faergeman O Olsson AG Thorgeirsson G Cholesterol lowering with simvastatin improves prognosis of diabetic patients with coronary heart disease. A subgroup analysis of the Scandinavian Simvastatin Survival Study (4S) Diabetes Care 1997 20 614 620 9096989 10.2337/diacare.20.4.614

[bib41] Olde Hartman TC Lucassen PL Van de Lisdonk EH Bor HH Van Weel C Chronic functional somatic symptoms: a single syndrome? Br J Gen Pract 2004 54 922 927 15588538 PMC1326111

[bib42] Fried TR Tinetti ME Iannone L Primary care clinicians' experiences with treatment decision making for older persons with multiple conditions Arch Intern Med 2011 171 75 80 20837819 10.1001/archinternmed.2010.318 PMC3021478

[bib43] Sinnott C Mc Hugh S Browne J Bradley C GPs' perspectives on the management of patients with multimorbidity: systematic review and synthesis of qualitative research BMJ Open 2013 3 e003610 10.1136/bmjopen-2013-003610 PMC3773648 24038011

[bib44] Luijks HD Loeffen MJ Lagro-Janssen AL Van Weel C Lucassen PL Schermer TR GPs' considerations in multimorbidity management: a qualitative study Br J Gen Pract 2012 62 e503 e510 22781998 10.3399/bjgp12X652373 PMC3381276

